# Flow cytometric characterization of acute leukemia reveals a distinctive “blast gate” of murine T-lymphoblastic leukemia/lymphoma

**DOI:** 10.18632/oncotarget.23410

**Published:** 2017-12-18

**Authors:** Zengkai Pan, Min Yang, Kezhi Huang, Guntram Büsche, Silke Glage, Arnold Ganser, Zhixiong Li

**Affiliations:** ^1^ Department of Hematology, Hemostasis, Oncology, and Stem Cell Transplantation, Hannover Medical School, Hannover, Germany; ^2^ Department of Hematology, Sun Yat-Sen Memorial Hospital, Sun Yat-Sen University, Guangzhou, China; ^3^ Institute of Pathology, Hannover Medical School, Hannover, Germany; ^4^ Institute of Laboratory Animal Science, Hannover Medical School, Hannover, Germany

**Keywords:** flow cytometry, T-ALL, blast gate, midostaurin, FLT3-ITD

## Abstract

Immunophenotypic analysis using multiparameter flow cytometry is an indispensable tool for diagnosis and management of acute leukemia. Mouse models have been widely used for medical research for more than 100 years and are indispensable for leukemia research. However, immunophenotypic analysis of murine leukemia was not always performed in published studies, and blast gating for isolation of blasts was shown only in very few studies. No systemic characterization of all types of murine acute leukemia in large cohorts by flow cytometry has been reported. In this study, we used flow cytometry to comprehensively characterize murine acute leukemia in a large cohort of mice. We found that murine T-lymphoblastic leukemia/lymphoma (T-ALL) exhibits a distinctive “blast gate” (CD45^bright^) with CD45/side scatter gating that differs from the “blast gate” (CD45^dim^) of human T-ALL. By contrast, murine B-lymphoblastic leukemia and acute myeloid leukemia show the same blast region (CD45^dim^) as human leukemia. Using blast cell gating, we for first time detected T-ALL development in FLT3-ITD knock-in mice (incidence: 23%). These leukemic cells were selectively killed by the FLT3 inhibitors crenolanib and midostaurin *in vitro*. These data suggest that FLT3-ITD plays a potential role in the pathogenesis of T-ALL and that FLT3-ITD inhibition is a therapeutic option in the management of patients with T-ALL. Our gating strategy for immunophenotypic analysis can be used for leukemogenesis and preclinical gene therapy studies in mice and may improve the quality of such analyses.

## INTRODUCTION

The immunophenotypic analysis of acute leukemia by multiparameter flow cytometry is a powerful tool for proper identification of lymphoid or myeloid lineage and is indispensable in modern diagnosis and management of acute leukemia [1]. CD45/side scatter (SS) gating for isolating blasts by flow cytometry was first proposed by Borowitz and Stelzer [2, 3] and is now widely used. Blasts in the classic “blast gate” (cBG) show CD45^dim^ in CD45/SS gating. However, other cells contaminate the cBG [4], and blasts may also occur in other gates. Mouse models have been widely used for medical research for more than 100 years and are indispensable for leukemic research. We and others [5–9] have suggested similar proposals for diagnosis of murine acute leukemia, including cytologic, histologic, and immunophenotypic analyses. In mouse models expressing transgene and/or marker genes such as enhanced green fluorescent protein (EGFP) in hematopoietic cells, leukemic cells may be easily isolated by flow cytometry [5, 10–13]. However, in many situations, for example in transgenic mice in which no marker gene was introduced into hematopoietic cells, blast cells may be difficult to distinguish from other cell types. So far, immunophenotypic analysis of murine leukemia was not always performed in published studies, and blast gating for isolation of blasts was shown only in very few studies. No systemic characterization of all types of murine acute leukemia in large cohorts by flow cytometry has been reported. In the present study, we analyze murine acute leukemia by flow cytometry in a large cohort of mice. We show that murine T-lymphoblastic leukemia/lymphoma (T-ALL) demonstrates a distinctive “blast gate” by CD45/SS gating. Our gating strategy for immunophenotypic analysis can be used for leukemogenesis and preclinical gene therapy studies in mouse models and may improve the quality of such analyses.

## RESULTS

### Murine T-ALL demonstrates a distinctive “blast gate” by CD45/SS gating

**Figure 1:**
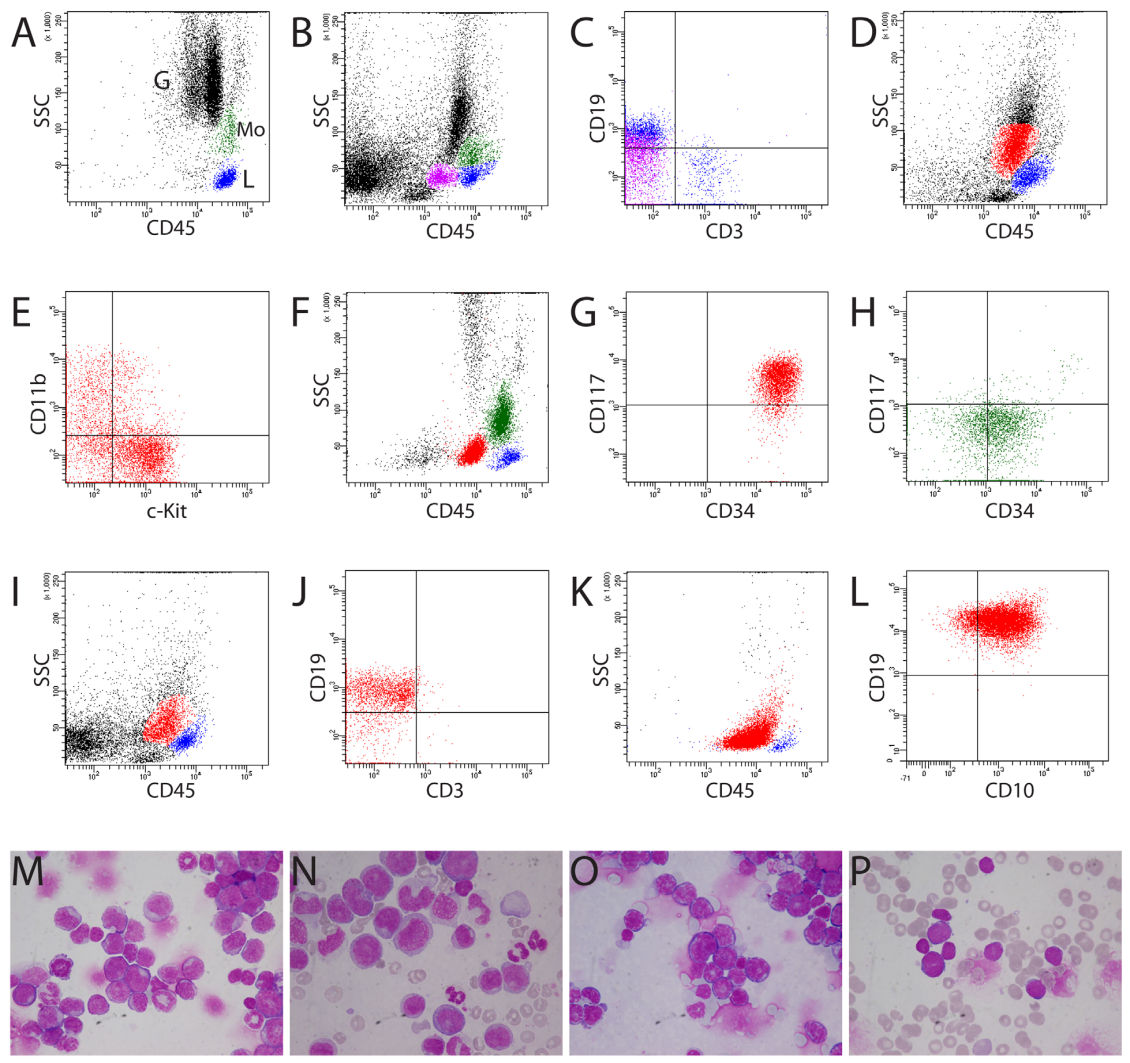
Clustering of normal bone marrow, AML, and B-lymphoblastic leukemia samples by CD45/SS gating **(A, B)** Characterization of normal human and murine bone marrow samples by CD45 gating, respectively. Blue population: T and B lymphocytes (L); green population: monocytes (Mo). Note the slightly different location of murine granulocytes (G) compared with human granulocytes because of less granularity in the cytoplasm of murine granulocytes (B). **(B, C)** In murine bone marrow, B lymphocytes (purple population) were also present in the classic “blast gate” (cBG, CD45^dim^ in CD45/SS gating). These cells had lower CD19 fluorescence intensity than B cells in the classic lymphocyte gate (blue population). **(D)** Blasts (red population) from murine AML (mouse #1356) with expression of c-Kit and CD11b **(E)** in the same location in the cBG as blasts with CD34 and c-Kit expression from patient TI with AML (red population) **(F, G)**. Monocytes from patient TI were heterogeneous and included mature and immature monocytes (CD34pos) **(H)**. **(I-L)** Blasts from both murine and human B-lymphoblastic leukemia located in the cBG (red populations) (I, J: mouse #1328, K, L: patient NS). (J) Murine B-lymphoblastic leukemia expressing CD19, not CD3. (L) Expression of CD19 and CD10 on the blasts from patient NS with common-ALL. **(M-P)** Cytology confirmed the presence of blasts in mice and patients (D-I). (N) Note blasts, immature and mature monocytes in patient TI (F-H). M = mouse #1356 (D, E); O = mouse #1328 (I, J); P = patient NS (K, L). M and O: bone marrow cytospins; N: blood smear; P: bone marrow smear.

**Figure 2:**
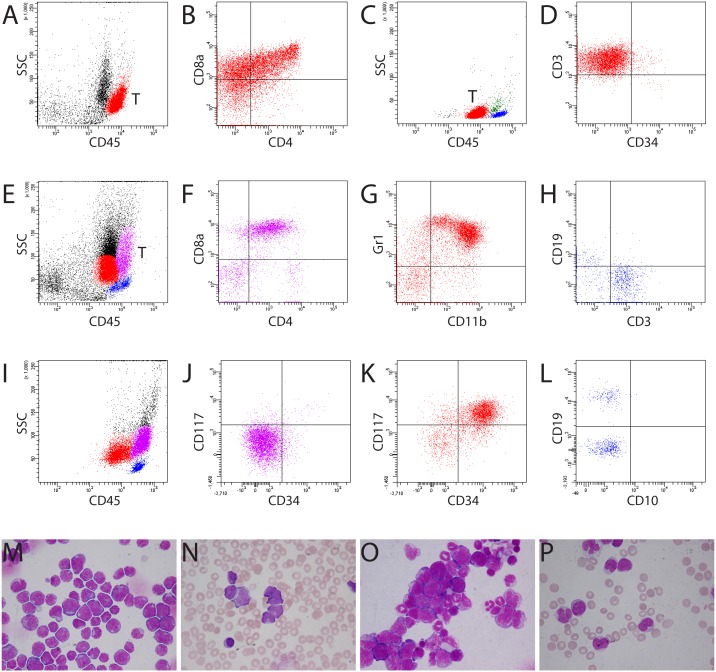
Different locations of blasts from murine and human T-ALL **(A)** Murine lymphoblasts from T-ALL (red population) located in a distinctive “blast gate” (aBG, CD45^bright^). T = T-lymphoblasts. **(B)** Murine lymphoblasts expressing CD4, CD8, and CD3 (data not shown). A, B: mouse #1364. **(C)** Human T-ALL blasts (red population) were present in the cBG (CD45^dim^). Blue population: lymphocytes; green population: monocytes. **(D)** Expression of cyCD3 on human blasts. C, D: patient LB. **(E)** Myeloblasts (red population) and T-ALL blasts (purple population) were located in different regions in the bone marrow of mouse #1329 (E-H). **(F)** T-ALL blasts expressing CD4/CD8. **(G)** Expression of CD11b and Gr1 on myeloblasts. **(H)** Mature lymphocytes expressing either CD3 or CD19. (I-L) Location of monoblasts in the aBG (purple population) from patient TK with AML M4. Note the slightly higher CD45 fluorescence intensity of monoblasts than of the mature lymphocytes **(I)**. Monoblasts were negative for CD117 or CD34 **(J)**, whereas myeloblasts (red population) expressed both CD117 and CD34 **(K)**. **(L)** Mature lymphocytes expressed CD19 (not CD10) or CD3 (data not shown). **(M, N)** Bone marrow cytospin and blood smear showing predominant murine and human lymphoblasts in mouse #1364 (A, B) and patient LB (C, D), respectively. **(O)** Bone marrow cytospin showing infiltration of myeloblasts and lymphoblasts in the bone marrow of mouse #1329 (E-H). **(P)** Blood smear showing blasts and promonocytes in patient TK with AML M4 (I-L). Blasts were positive for Sudan Black B and non-specific esterase (Supplementary Figure 3).

We performed experiments for leukemogenesis and preclinical gene therapy studies in more than 3,000 mice, including transplantation of retrovirally modified hematopoietic cells into C57Bl/6J and C3H/HeJ mice, serial transplantation of murine leukemic cells, and studies of transgenic and knock-in mice [5, 10–17]. At least 500 animals developed acute leukemia. A diagnosis of leukemia was established generally based on cytologic, histologic, and/or immunophenotypic findings. Consistent with clustering of human bone marrow cells in CD45/SS gating (Figure 1A), bone marrow cells from healthy mice and mice without hematological malignancies showed similar distribution of cell populations (Figure 1B), except for the location of some B lymphocytes in the CD45^dim^ cBG in most analyzed cases (n = 22) (Figures 1C and Supplementary Figure 1). Contamination of non-blast cells in the cBG has been reported in patients without acute myeloid leukemia (AML) [4]. Blasts from mice with AML (non-monocytic) (n = 31) and B-lymphoblastic leukemia (n = 8) were in the same location in the cBG as blasts from patients with acute leukemia (Figures 1D-1L). Cytological assessment confirmed the presence of acute leukemia in these mice and patients (Figures 1M-1P). While the cBG for human AML and B-lymphoblastic leukemia was clearly separated from granulocytes and monocytes (Figures 1F and 1K), the cBG for murine AML and B-lymphoblastic leukemia was sometimes difficult to set because of the close proximity of granulocytes and monocytes (Figure 1B). Interestingly, blasts from murine T-ALL were located in a gate with the highest CD45 fluorescence intensity (CD45^bright^) (Figures 2A-2P), whereas blasts from patients with T-ALL were exclusively present in the cBG (Figures 2C and 2D). This distinctive “blast gate” (CD45^bright^) (Figure 2A) was reproducibly seen for T-ALL in all analyzed mice (n = 74) from different experiments addressing the role of several genes in the leukemogenesis, for example, ΔTRKA [15], TRKA/NGF [13], TRKB/BDNF [12], and p53 (in p53 knock-out and p53/FLT3 double transgenic mice). This blast gate was consistent for T-ALL cells from thymus, spleen, liver, and bone marrow, and for T-ALL with different phenotyping (e.g. CD4/CD8 double positive, CD4 or CD8 single positive, and CD4/CD8 double negative) (data not shown). We proposed this distinctive “blast gate” of T-ALL as an alternative “blast gate” (aBG). Interestingly, this blast region was also seen in Pten-null T-ALL [18]. Moreover, normal T precursor cells isolated from thymus of wild type (WT) mice (n = 2) and one mouse without hematological malignancies were also located in this aBG as T-ALL (Supplementary Figure 1). Importantly, infiltration of T-ALL and AML cells was observed in the bone marrow of some mice, demonstrating different locations of myeloblasts and lymphoblasts in the same sample (Figures 2E-2H). Moreover, we mixed T-ALL cells with AML cells and other cell populations from different mice, and flow cytometric analysis confirmed the same clustering of T-ALL cells (Supplementary Figure 2 and data not shown). Of note, none of the human T-ALL blasts were located in the aBG in a cohort of patients with acute leukemia (n > 200) including ALL (n = 21), but we observed monoblasts in the aBG from some patients with AML M4/5 (Figures 2I-2L) or in the monocyte gate (Figures 1F and 1H) as reported by Gorczyca [19]. Acute monocytic leukemia is rare in mice. Blasts from the mouse with acute monocytic leukemia in our cohort [16] were also found in the aBG (Supplementary Figure 4).

### T-ALL development in FLT3-ITD knock-in mice

**Figure 3:**
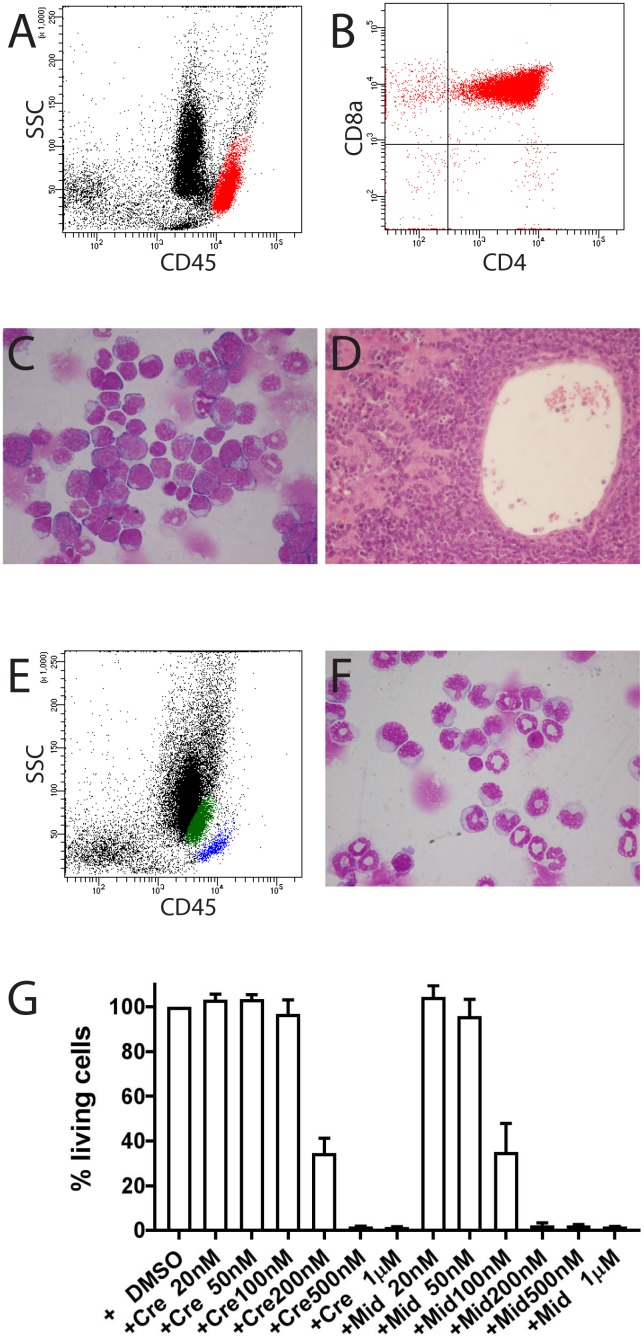
Development of T-ALL in mouse FLT3#457 (FLT3 ITD/ITD) **(A-D)**. (A) Location of T-lymphoblasts (red population) from bone marrow in the aBG (CD45^bright^) with expression of CD4 and CD8a (B). (C) Bone marrow cytospin showing infiltration of blasts. (D) Liver section showing strong infiltration of blasts. **(E, F)** CMML in mouse #1382. (E) Flow cytometric analysis showing increased monocytes in bone marrow (green population). Blue population: T and B lymphocytes. (F) Bone marrow cytospin confirming increased monocytes in bone marrow. **(G)** Leukemic cells from mouse FLT3#457 were sensitive to treatment with FLT3 inhibitors crenolanib and midostaurin. Results are presented as the average percentage of living cells in the presence of inhibitors (100% value derived from DMSO control). Representative results presented are the mean ± SD (error bars) of three independent experiments. Similar results were also observed with leukemic cells from mouse #1361. Cre = crenolanib; Mid = midostaurin.

Using this gating strategy, we performed immunophenotypic analysis in selected FLT3-ITD knock-in mice, in which an 18-bp ITD mutation was inserted into the juxtamembrane domain of the FLT3 gene [20]. This allows physiological expression of FLT3-ITD from its endogenous promoter and better understanding of the role of FLT3-ITD in leukemogenesis. Homozygous FLT3-ITD/ITD (ITD/ITD) mice develop myeloproliferative disease after a long latency [20, 21]. We found T-ALL in 7 out of 30 (23%) ITD/ITD mice (Figures 3A-3D), in which infiltration of tumor cells was observed in different organs. Other mice developed a (Chronic myelomonocytic leukemia=CMML)-like disease or AML (n=1) as reported (Figures 3E and 3F) [20, 21]. To the best of our knowledge, our report is the first report of T-ALL in FLT3-ITD knock-in mice. Together with an early report showing ALL development in FLT3-ITD transgenic mice [22], our data suggest a potential role of FLT3-ITD in the pathogenesis of T-ALL. Accordingly, FLT3 mutations have been reported in patients with T-ALL and B-lymphoblastic leukemia [23], particularly among children with ALL (incidence: up to 25%) [24, 25]. Moreover, leukemic cells from mice with T-ALL were sensitive to FLT3 inhibition (Figure 3G). Crenolanib and midostaurin almost completely killed the cells at 500 nM and 200 nM concentrations, respectively, similar to the concentration (crenolanib) or well below the concentration (midostaurin) safely achieved in humans [26–28]. Our data indicate that crenolanib and midostaurin might be effective in treating the subset of T-ALL patients with FLT3-ITD.

## DISCUSSION

Mouse models facilitate our understanding of the molecular mechanisms for leukemia development. However, immunophenotypic analysis of leukemic cells is not well investigated, and blast cell gating for isolation of blasts has been performed so far only in very few studies. Guo et al. [18, 29] described the blast gating for T-ALL in an early study. However, they analyzed only leukemia in *Pten*-mutant mice, and no murine B-lymphoblastic leukemia was characterized. No report has confirmed their finding. Whether the blast cell gating can be applied to other murine leukemias is not clear. In the present study of a large cohort of mice, we extended the finding of Guo et al., and confirmed the same blast gating for T-ALL in different models. Moreover, we analyzed other types of murine malignancies, including B-lymphoblastic leukemia, monocytic leukemia, and lymphoblastic lymphoma, that were not addressed by Guo et al. We demonstrate a distinctive “blast gate” (aBG, CD45^bright^) of murine T-ALL derived from different mouse models that is different from the blast gate of human T-ALL, whereas other murine leukemia (AML and B-lymphoblastic leukemia) show the same blast region as human leukemia. Use of cBG (CD45^dim^) for immunophenotypic analysis in cases of murine T-ALL can lead to a wrong diagnosis, particularly in mice without enlargement of the thymus. With the use of blast gating (i.e. aBG), we found T-ALL development in FLT3-ITD knock-in mice (incidence: 23%) that was not reported before in the same models, probably due to the use of different gating. Without the correct blast gating, we would have also overlooked some cases of T-ALL in our model. Pitfalls in the use of the cBG for blast analysis have been also reported in patients without AML [4].

The reason for this aBG of murine T-ALL is unknown and needs to be determined. CD45 is shown to regulate phosphorylation of kinases such as SRC and JAK family kinases and might have a tumor suppressor role in T-ALL [30, 31]. However, high CD45 expression on leukemic blasts was associated with a poor prognosis in children with precursor B-cell and T-cell ALL, mainly due to a higher cumulative incidence of relapse [32]. In our cohort, mice with T-ALL seemed to have shorter survival than mice without T-ALL. However, in some instances an enlarged thymus might also have contributed to shorter survival of some mice.

FLT3 plays a critical role in the maintenance of hematopoietic homeostasis. In humans, FLT3 is expressed on hematopoietic stem cells, and FLT3 signaling prevents apoptosis in stem cells and progenitors and induces proliferation and maintenance of stem/progenitor cells [33]. Mutated FLT3 has been identified in approximately 30% of AML patients, making it one of the most common mutations with prognostic implications observed in this disease. Frequently, the mutation is an in-frame internal tandem duplication (ITD) in the juxtamembrane region or a point mutation in tyrosine kinase domain-1 that results in constitutive activation of FLT3. A recently published trial that included 717 patients with previously untreated FLT3+ AML demonstrated a statistically significant improvement in overall survival (OS) for patients treated with midostaurin (targeting FLT3-ITD) compared with those on the placebo-containing arm (HR 0.77, *P* = 0.016) [34]. This led to recent FDA approval of midostaurin as frontline therapy for adult patients with newly diagnosed AML. In this study, our data suggest a potential role of FLT3-ITD in the development of T-ALL. We showed that murine T-ALL induced by FLT3-ITD is highly sensitive to treatment with FLT3 inhibitors, including midostaurin. Early studies demonstrated that ALL samples from patients with FLT3 mutations or high level of FLT3 expression were selectively killed by FLT3 inhibition [24]. These data suggest that targeting FLT3-ITD might be a treatment option for ALL patients with FLT3 mutations or high FLT3 expression.

In conclusion, our study is the first comprehensive study that characterizes all common types of murine acute leukemia in a large cohort of mice by flow cytometry. Careful immunophenotypic analysis of leukemic cells with the right blast gating may improve the quality of mouse experiments. Because both murine and human blasts can be in both blast gates (cBG with CD45^dim^ and the aBG with CD45^bright^), our data highlight careful isolation of blasts in acute leukemia by flow cytometry, and the proposed aBG is thus helpful for making appropriate diagnoses of T-ALL in mouse models. Moreover, for clinical diagnosis and management of patients with acute leukemia, it should be aware that leukemic blasts can be also isolated from the aBG with CD45^bright^.

## MATERIALS AND METHODS

### *In vivo* tumorigenesis assays

Retroviral transductions of murine hematopoietic cells and *in vivo* tumorigenesis assays have been described [5, 10–16, 35]. We purchased FLT3-ITD knock-in (KI) mice from the Jackson Laboratory, in which an 18-bp ITD mutation was inserted into the juxtamembrane domain of the FLT3 gene. The mice with C57BL/6 background were originally established in the Gilliland´s laboratory [20]. Upon arrival at the Jackson Laboratory, the mice were crossed to C57BL/6J mice at least once to establish the colony. After arrival at our animal facility, the mice were crossed to C57BL/6J at least three times in order to obtain a high C57BL/6J background. FLT3-ITD mice were crossed to p53 knock-out mice to generate double transgenic mice. Animal experiments were approved by the local ethical committee and performed according to their guidelines.

### Tumor phenotyping

At the end point analysis, mice were macroscopically examined for pathological abnormalities during dissection. Enlarged organs were weighed. Bone marrow, spleen, liver, skin, gut, kidney, lung, brain and thymus were fixed in a buffered 4% formalin solution and embedded in Paraplast Plus (Kendall, Mansfield, MA, USA). Sections were routinely stained with hematoxylin and eosin. Blood cell counts were measured by an automatic analyzer (ABC Counter, Scil, Viernheim, Germany). Cytological and histological examinations were performed as previously described [12].

### Flow cytometric analyses

Flow cytometric analyses were performed by use of whole murine bone marrow/spleen cells and patient samples after Ficoll separation (thus lower erythrocytes and debris in patient samples; see e.g. Figure 1A). Murine cells were stained with fluorescein isothiocyanate (FITC)-conjugated, R-phycoerythrin (PE)-conjugated, allophycocyanin (APC)-conjugated, APC-eFluor 780-conjugated, Percp-cy5.5-conjugated, or PE-Cy7-conjugated antibodies, including CD45, CD34, CD117, CD11b, Gr-1, Ter119, CD71, F4/80, CD4, CD8a, CD3, CD19, B220, CD44, and CD25 (from eBioscience or Biolegend). Human cells were stained with fluorescein isothiocyanate (FITC)-conjugated, R-phycoerythrin (PE)-conjugated, allophycocyanin (APC)-conjugated, APC-H7-conjugated, Percp-cy5.5-conjugated, or PE-Cy7-conjugated antibodies, including CD45, CD34, CD117, myeloperoxidase, CD13, CD14, CD15, CD33, CD61, CD235a (glycophorin A), HLA-DR, CD3, CD4, CD8, CD1a, CD2, CD5, CD7, CD8, Tdt, TCR α/β, TCR γ/δ, CD19, CD10, CD20, CD20, CD79a, kappa, lamda, and IgM (from BD Biosciences or Biolegend). Intracellular staining was performed using an IntraPrep Permeabilization Reagent Kit according to the manufacturer's protocol (Beckman Coulter, Marseille, France). The cells stained with antibodies were analyzed by flow cytometry either on an FACSCalibure or FCASCanto (BD Bioscience). Generally, atleast 10,000 cells per tube were measured and analyzed. Leukemic blasts were analyzed on CD45/SSC plots as previously described [2, 3].

### Apoptosis assay

Leukemic cells were cultured in the presence of inhibitors for 48 hours before apoptosis analysis. Cell viability was analyzed using the Annexin-V assay (BD Pharmingen, Heidelberg, Germany) on an FCASCanto. We generally measured and analyzed at least 10,000 cells per tube. Annexin V^+^/PI^-^ and Annexin V^+^/PI^+^ cells are considered cells in the early stage of apoptosis and the late-stage of apoptosis, respectively. The inhibitors crenolanib and midostaurin were purchased from Selleckchem (Houston, TX).

## SUPPLEMENTARY MATERIALS FIGURES


