# Supplementary material for: Flow cytometric characterization of acute leukemia reveals a distinctive “blast gate” of murine T-lymphoblastic leukemia/lymphoma
Source: Oncotarget. 2017 Dec 18;9(2):2320–8. doi: 10.18632/oncotarget.23410 (PMC5788642; doi:10.18632/oncotarget.23410)
Supplement: Supplementary file 1 [file oncotarget-09-2320-s001.pdf]

Flow cytometric characterization of acute leukemia reveals a distinctive "blast gate" of murine T-lymphoblastic leukemia/ lymphoma

SUPPLEMENTARY MATERIALS

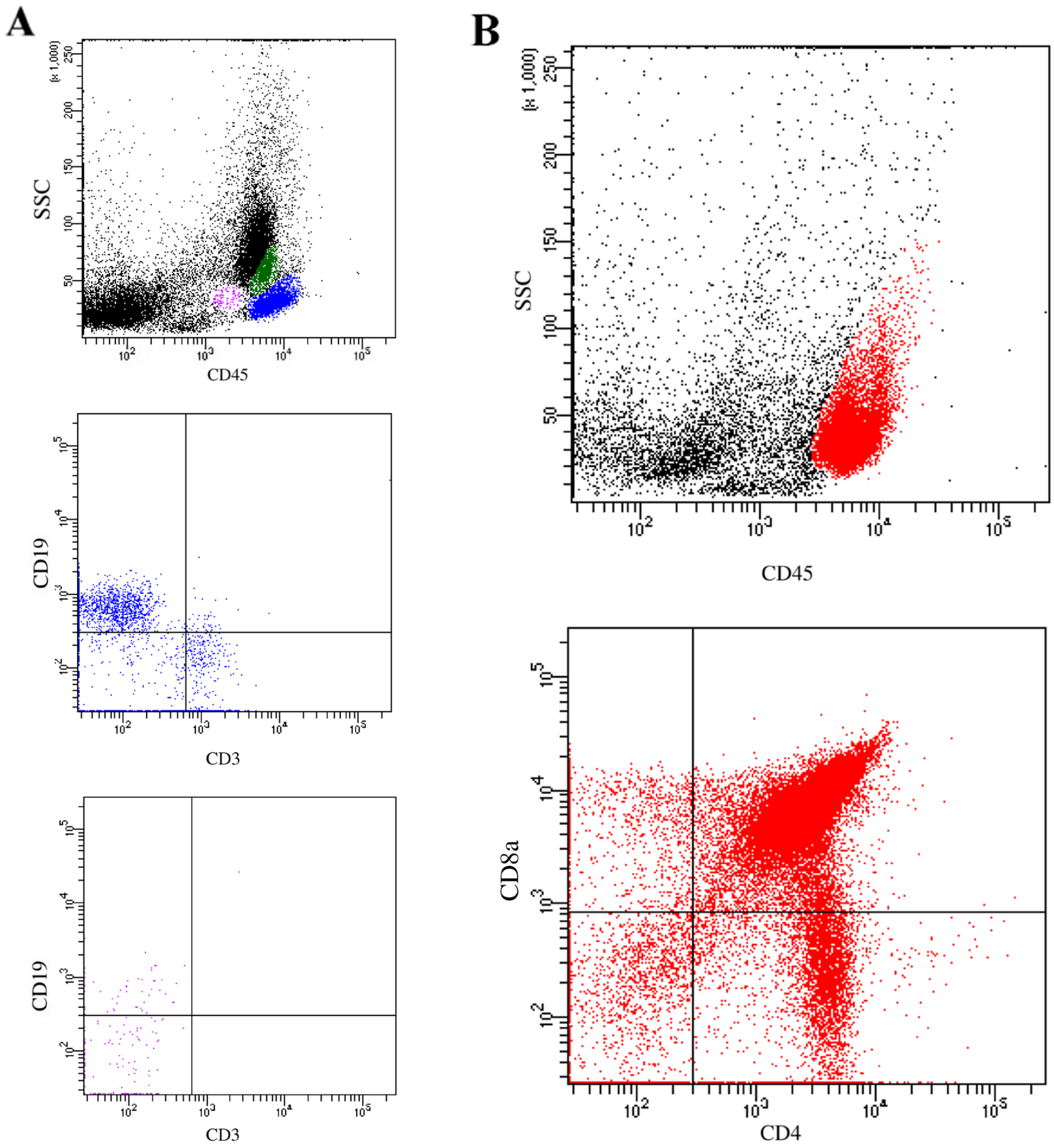

Supplementary Figure 1: Distribution of normal bone marrow cells and thymocytes by flow cytometric analysis. (A) Mouse #1395 without hematological malignancies showed less contamination of B cells (purple population) in the cBG than wild type (WT) mice (Figure 1B). Blue population: T and B lymphocytes; green population: monocytes. **(B)** Thymocytes from WT mice were located in the aBG as T-ALL cells. Note presence of double negative ($CD4^-/CD8^-$), double positive ($CD4^+/CD8^+$), and single CD4 or CD8 positive cells.

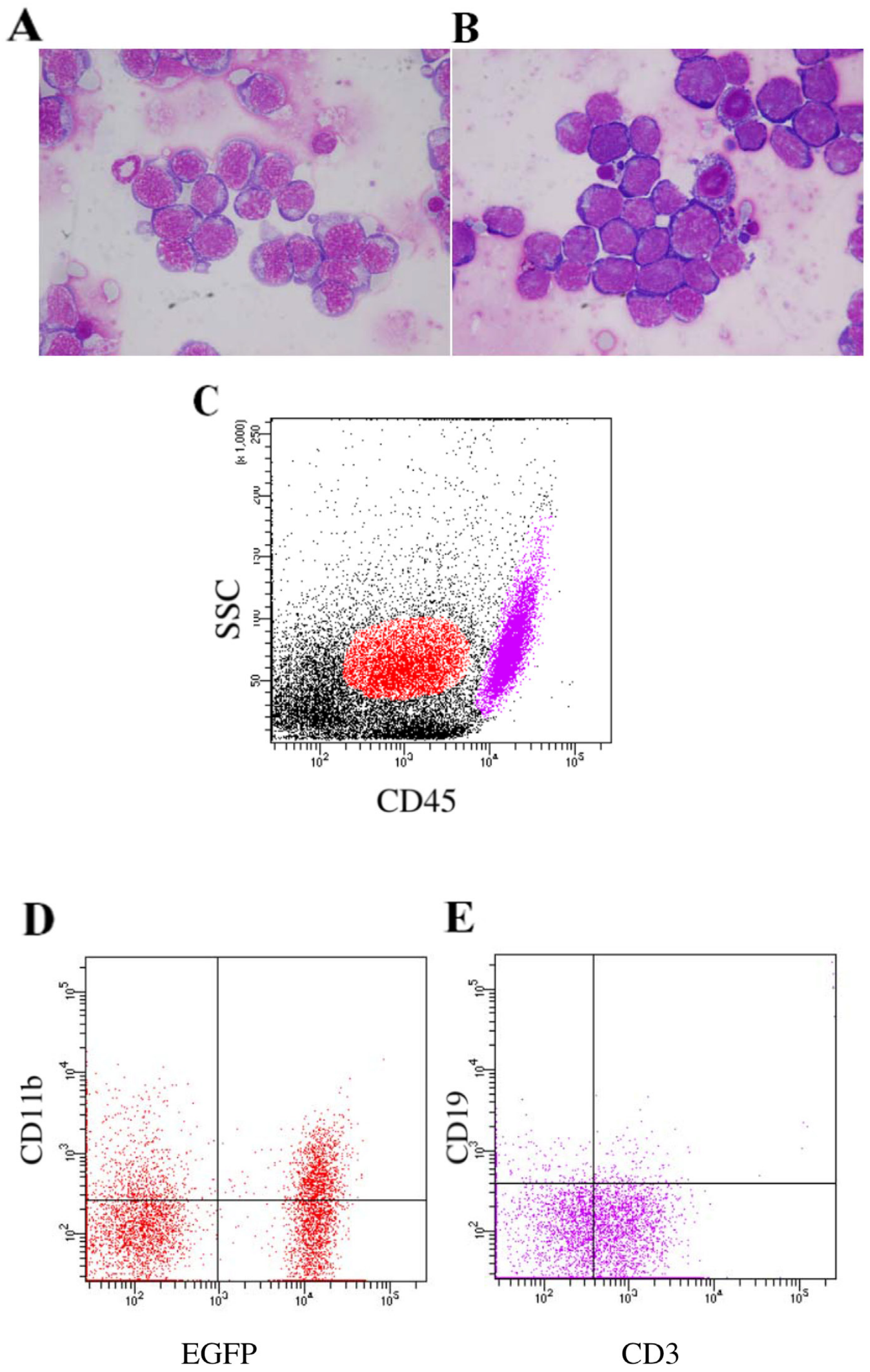

Supplementary Figure 2: AML cells from C3H/HeJ mouse #1294 **(A)** were mixed with T-ALL cells from C57BL/6J mouse #1414 **(B). (C)** Flow cytometric analysis showed different clustering of AML cells (in the cBG, red population) and T-ALL cells (in the aBG, purple population) by CD45/SSC gating. **(D)** AML cells expressed CD11b and marker gene EGFP. **(E)** T-ALL cells were positive for CD3 and CD8 (data not shown).

A

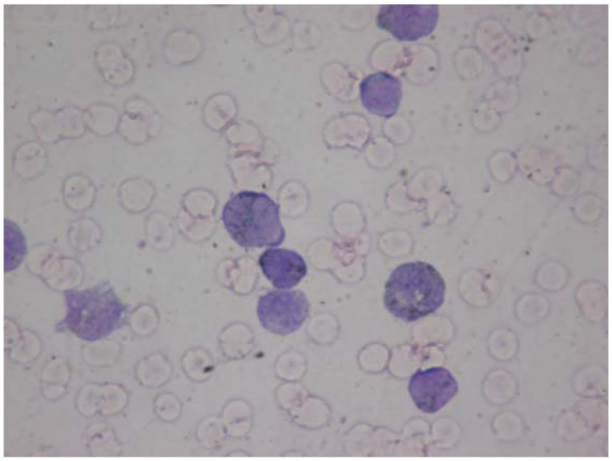

B

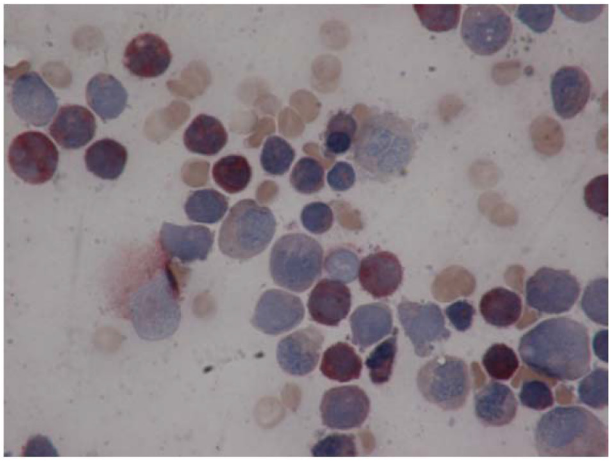

Supplementary Figure 3: Blasts from patient TK (Figure 2 I, P) were positive for Sudan Black B **(A)** and non-specific esterase **(B).**

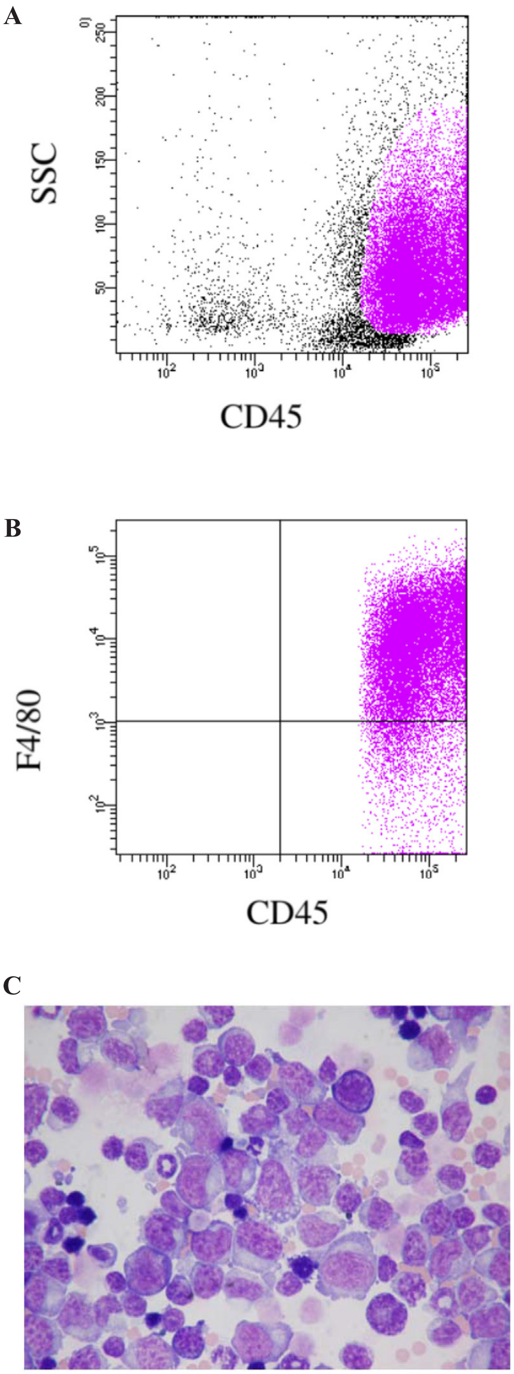

Supplementary Figure 4: Development of monocytic leukemia in mouse #597. (A) Monoblasts (purple population) from spleen located in the aBG (CD45^{bright}). **(B)** Monoblasts expressing F4/80, CD11b, and c-Kit (data not shown). **(C)** Spleen cytosine showing infiltration of monoblasts.
